# Longitudinal association of SGLT2 inhibitors and GLP-1RAs on falls in persons with type 2 diabetes

**DOI:** 10.1038/s41598-025-91101-0

**Published:** 2025-03-17

**Authors:** Yasuhiro Suzuki, Hiroaki Suzuki, Kazushi Maruo, Takaaki Matsuda, Yuki Murayama, Yoko Sugano, Yoshinori Osaki, Hitoshi Iwasaki, Motohiro Sekiya, Yasushi Hada, Hitoshi Shimano

**Affiliations:** 1 Department of Endocrinology and Metabolism, Faculty of Medicine, University of Tsukuba, 1-1-1 Tennodai, Tsukuba, Ibaraki 305-8575 Japan; 2 Biomedical Science and Engineering Research Center, Hakodate Medical Association Nursing and Rehabilitation Academy, Hakodate, Hokkaido Japan; 3 Department of Rehabilitation Medicine, University of Tsukuba Hospital, Tsukuba, Ibaraki Japan; 4 Faculty of Systems, Information and Engineering, University of Tsukuba, Tsukuba, Ibaraki Japan; 5 Department of Food and Health Sciences, Faculty of Human Life Sciences, Jissen Women’s University, 4-1-1 Osakaue, Hino, Tokyo, 191-8510 Japan; 6 Department of Biostatistics, Institute of Medicine, University of Tsukuba, Tsukuba, Ibaraki Japan; 7 Tsukuba Clinical Research and Development Organization (T-CReDO), University of Tsukuba, Tsukuba, Ibaraki Japan; 8 Department of Endocrinology and Metabolism, University of Tsukuba Hospital, Tsukuba, Ibaraki Japan; 9 Department of Rehabilitation Medicine, Institute of Medicine, University of Tsukuba, Tsukuba, Ibaraki Japan; 10 International Institute for Integrative Sleep Medicine (WPI-IIIS), University of Tsukuba, Tsukuba, Ibaraki Japan; 11 Life Science Center of Tsukuba Advanced Research Alliance (TARA), University of Tsukuba, Tsukuba, Ibaraki Japan; 12 Japan Agency for Medical Research and Development-Core Research for Evolutional Science and Technology (AMED-CREST), Chiyoda-ku, Tokyo, Japan

**Keywords:** Drug discovery, Physiology, Diseases, Endocrinology, Health care, Medical research, Risk factors

## Abstract

Low lean body mass increases fall risk. Some diabetes medications, specifically SGLT2 inhibitors and GLP-1RAs, can cause muscle and body mass loss. This study assessed their association on falls in type 2 diabetes patients. An annual fall survey was conducted for up to 5 years on individuals with type 2 diabetes admitted to our department. Fall risk factors were identified using discrete-time survival analysis. The study observed 471 participants over a median period of 2 years. The participants had a median age of 64 years, with a fall incidence rate of 17.1 per 100 person-years. Independent fall predictors identified were fall history, SGLT2 inhibitor use, and age. The odds ratios (95% confidence intervals) for using SGLT2 inhibitors only, GLP-1RAs only, and both combined were 1.80 (1.10–2.92), 1.61 (0.88–2.84), and 2.89 (1.27–6.56), respectively. SGLT2 inhibitor use was an independent risk factor for falls, while GLP-1RAs’ effects were not statistically significant. However, the combined use of SGLT2 inhibitors and GLP-1RAs significantly increased the risk of falls. Therefore, it is important to consider this risk when prescribing these medications to people with type 2 diabetes.

## Introduction

Falls and related injuries can occur during walking or other physical activities at any age and are a significant health and socioeconomic burden, causing distress, physical disability and other medical conditions, hospitalization, institutionalization, and even death^1,2^. In particular, ~ 17 million person-years of life were lost because of falls in 2017^1^, and fall-related costs account for ~ 1% of healthcare spending in developed countries^3^. Major fall risk factors include a history of falls, balance and visual impairment, muscle weakness, certain medications, gait problems, cognitive decline, and diabetes^4,5^.

Elderly individuals with diabetes are 1.5–3 times more likely to experience falls than those without diabetes^5^. Previously reported risk factors for falls in persons with diabetes include diabetic neuropathy, diabetic retinopathy, elevated cystatin C levels, insulin or sulfonylurea use, and decreased grip strength, knee extension, and ankle dorsiflexor muscle strength^6–10^. A prospective cohort study on risk factors for falls in persons with type 2 diabetes revealed a positive correlation between severe hypoglycemia and fall prevalence (hazard ratio = 2.23) and suggested that the increased fall incidence related to insulin or sulfonylureas use can be attributed to the ability of these medications to induce hypoglycemia^11^. Previously, we used machine learning to identify significant fall risk factors in functionally independent elderly and nonelderly individuals with type 2 diabetes as grip strength, fasting serum C-peptide level, knee extension muscle strength, ankle dorsiflexor muscle strength, and proliferative diabetic retinopathy; however, insulin and sulfonylurea treatments were not identified as significant fall risk factors^12^. The emergence of numerous blood glucose–lowering medications has led to a decrease in the use of sulfonylureas and an increase in the use of dipeptidyl peptidase-4 inhibitors, glucagon-like peptide-1 receptor agonists (GLP-1RAs), and sodium-glucose cotransporter 2 (SGLT2) inhibitors in combination therapies^13^. Compared with insulin therapy alone, insulin therapy coupled with the use of these medications was reported to achieve better blood glucose control without increasing the risk of hypoglycemia^14–16^. The coadministration of basal insulin with oral glucose-lowering medications or GLP-1RAs poses a lower risk of hypoglycemia than basal-bolus therapy while retaining the efficiency of the latter in managing blood glucose levels^17,18^. Given the decrease in the risk of hypoglycemia with the advances in diabetes pharmacotherapy, insulin and sulfonylurea use may no more be an important fall risk factor. However, GLP-1RAs and SGLT2 inhibitors are known to reduce body weight, affecting both fat and lean body masses^19^, and may therefore lead to muscle weakness and increase the risk of falls. To examine the hitherto unexplored correlation between the administration of these drugs and fall incidence, we herein examined the effects of GLP-1RAs and SGLT2 inhibitors on falls in elderly and nonelderly persons with type 2 diabetes, and assessed the corresponding fall risks.

## Results

**Fig. 1:**
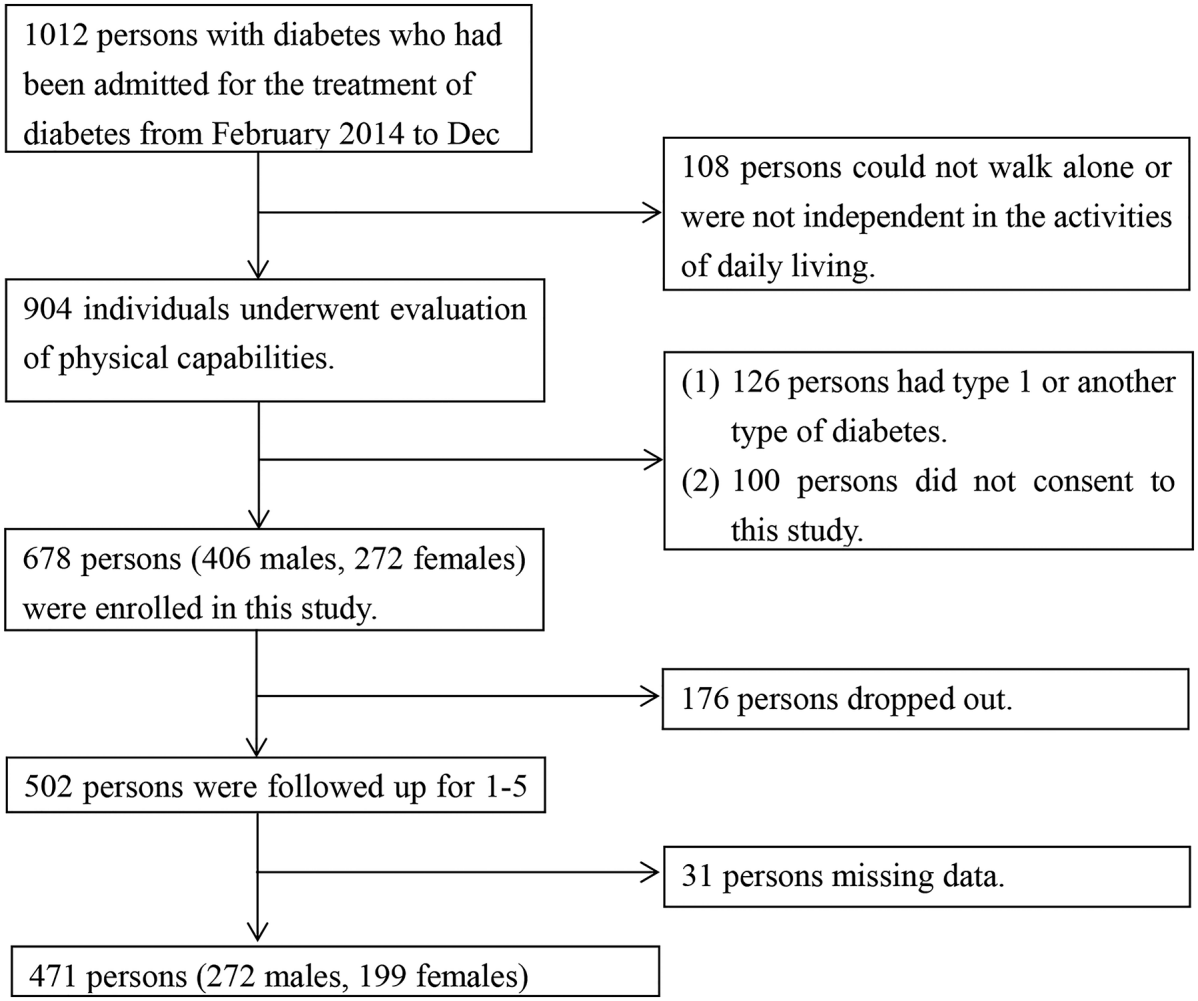
Flowchart of selection of study participants.

In total, 678 persons were enrolled in the study. Of these, 471 were included in the analysis after excluding participants that did not return questionnaires or provide data on weight at the first year postdischarge (Fig. 1). The participants (272 males and 199 females: 471 in total) had a median age of 63 (51–71) years. The median follow-up period was 2 (1–3) years, corresponding to a total of 1,013 person-years of observation. The number of individuals who reported at least one fall after discharge was 173, corresponding to a fall incidence rate (number of falls/total person-years of observation) of 17.1 per 100 person-years. Fifteen individuals experienced fractures due to falls. The follow-up rates of the fall survey (respondents/survey recipients) at the first, second, third, fourth, and fifth years after discharge were 69% (471/678), 55% (265/482), 44% (161/367), 39% (107/274), and 29% (62/192), respectively. The corresponding follow-up rates for the weight survey were 69% (465/678), 53% (256/482), 41% (152/367), 38% (104/274), and 29% (56/192), respectively. The changes in body weight in the first, second, third, fourth, and fifth years after discharge were − 0.7 (− 3.2 to 1.7), 0.2 (− 3.0 to 3.1), 0.0 (− 3.2 to 2.5), − 0.3 (− 4.0 to 2.9), and − 0.2 (− 3.6 to 3.5) kg, respectively.

Tables 1 and 2 present the characteristics of participants who responded to the survey one 1 year after discharge. Among the participants taking GLP-1RAs, 42 were on dulaglutide, 20 were on liraglutide, 6 were on semaglutide, 3 were on exenatide extended-release (exenatide XR), and 3 were on lixisenatide. To examine the risk factors for postdischarge falls, we carried out discrete-time survival analysis using fall history at admission, gender, age, height, BMI, grip strength, fasting serum CPR level, knee extension muscle strength, ankle dorsiflexion muscle strength, presence of proliferative retinopathy, glucose-lowering drugs used at discharge, and postdischarge weight change as covariates. Fall history at admission, SGLT2 inhibitor intake, and age were identified as independent predictors for falls in all models, and gender was additionally identified as such for Model 1. In all models, GLP-1RA intake was not a significant risk factor, and no significant interaction between SGLT2 inhibitor and GLP-1RA intakes was observed. The C-indices of Models 1, 2, 3, and 4 were 0.682, 0.606, 0.593, and 0.550. The odds ratios (95% confidence intervals) for falls in the best-performing model (Model 1) were as follows: Fall history = 2.26 (1.57–3.26), SGLT2 inhibitor intake = 1.80 (1.10–2.92), gender (female) = 1.73 (1.03–2.89), age = 1.02 (1.01–1.04), and GLP-1RA intake = 1.61 (0.88–2.84) (Table 3).

**Table 1:** Participant characteristics, including administered medications.

Parameter	All *n* = 471	Neither *n* = 327	SGLT2 inhibitors *n* = 74	GLP-RAs *n* = 53	Combination *n* = 17
Age (year)	63 (51–71)	64 (52–71)	61 (46–69)	59 (52–72)	65 (48–69)
Female gender (count/%)	199/42.3	143/43.7	27/36.5	21/39.6	8/47.1
Duration of diabetes (year)	10 (2–17)	9 (2–16)	10 (2–16)	15 (4–20)	14 (6–20)
Fall history at admission (count/%)	100/21.2	73/22.3	16/21.6	9/17.0	2/11.8
Height (m)	1.62 (1.55–1.69)	1.61 (1.54–1.68)	1.64 (1.57–1.72)	1.63 (1.57–1.68)	1.59 (1.53–1.69)
Body weight (kg)	68.8 ± 16.5	66.6 ± 15.5	74.9 ± 16.1	73.8 ± 20.4	69.6 ± 15.1
Body mass index (kg/m^2^)	26.1 ± 5.3	25.4 ± 4.8	27.7 ± 5.2	27.8 ± 7.2	26.6 ± 5.3
Fasting plasma glucose (mM)	8.7 (7.2–10.6)	8.6 (7.0–10.6)	8.7 (7.3–10.4)	9.8 (8.1–11.6)	8.3 (7.3–9.0)
Fasting serum C-peptide (nM)	0.5 (0.3–0.8)	0.5 (0.3–0.7)	0.7 (0.4–0.9)	0.6 (0.3–0.9)	0.5 (0.4–0.8)
HbA1c (%)	9.6 ± 1.9	9.7 ± 2.0	9.5 ± 1.8	9.8 ± 1.8	9.3 ± 1.6
HbA1c (mmol/mol)	82 ± 21	82 ± 21	81 ± 20	84 ± 20	78 ± 18
Total cholesterol (mM)	4.9 ± 1.1	4.9 ± 1.1	4.7 ± 0.9	5.0 ± 1.1	4.8 ± 0.9
Low-density lipoprotein cholesterol (mM)	2.9 ± 0.9	2.9 ± 0.9	2.8 ± 0.8	3.0 ± 0.8	2.8 ± 0.8
High-density lipoprotein cholesterol (mM)	1.2 (1.0–1.4)	1.2 (1.0–1.5)	1.2 (1.0–1.4)	1.2 (1.0–1.4)	1.1 (0.9–1.3)
Triglycerides (mM)	1.4 (1.0–2.0)	1.4 (1.0–1.9)	1.6 (1.1–2.1)	1.5 (1.1–2.3)	1.7 (1.2–2.6)
Creatinine (µΜ)	69.9 ± 42.6	68.2 ± 45.3	68.8 ± 23.3	76.0 ± 41.0	87.4 ± 53.8
Estimated glomerular filtration rate (mL/(min × 1.73 m^2^))	76 (62–97)	80 (64–98)	73 (61–92)	73 (60–95)	72 (43–91)
Urinary albumin excretion (mg/day)	10.5 (5.4–38.3)	10.4 (5.5–35.0)	11.6 (5.1–62.4)	11.2 (5.4–55.5)	10.6 (6.4–33.3)
Diabetic polyneuropathy (count/%)	120/25.5	83/25.4	15/20.3	14/26.4	8/47.1
Impaired vibratory sensation (count/%)	120/25.5	83/25.4	18/24.3	13/24.5	6/35.3
Cardiac autonomic neuropathy (count/%)	187/39.7	132/40.4	26/35.1	24/45.3	5/29.4
Nephropathy (count/%)	147/31.2	95/29.1	24/32.4	23/43.4	5/29.4
Retinopathy (count/%)	34/7.2	24/7.3	5/6.8	4/7.5	1/5.9
Brachial-ankle pulse wave velocity (cm/s)	1671 ± 431	1677 ± 450	1644 ± 393	1663 ± 409	1703 ± 282
Coronary artery disease (count/%)	35/7.4	20/6.1	7/9.5	6/11.3	2/11.8
Stroke (count/%)	26/5.5	19/5.8	4/5.4	3/5.7	0/0
Peripheral arterial disease (count/%)	12/2.5	7/2.1	2/2.7	3/5.7	0/0
Medications at discharge					
Sulfonylureas (count/%)	51/10.8	36/11.0	5/6.8	8/15.1	2/11.8
Glinides (count/%)	13/2.8	7/2.1	3/4.1	3/5.7	0/0
Biguanides (count/%)	284/60.3	183/56.0	56/75.7	34/64.2	11/64.7
Sodium-glucose cotransporter 2 inhibitors (count/%)	91/19.3	0/0	74/100	0/0	17/100
α-glucosidase inhibitors (count/%)	33/7.0	24/7.3	5/6.8	2/3.8	2/11.8
Thiazolidinedione (count/%)	28/5.9	8/2.4	8/10.4	9/17.0	3/17.6
Dipeptidyl peptidase-4 inhibitors (count/%)	209/44.4	164/50.2	40/54.1	5/9.4	0/0
Glucagon-like peptide-1 receptor agonists (count/%)	70/14.9	0/0	0/0	53/100	17/100
Insulin (count/%)	242/51.4	167/51.1	41/55.4	28/52.8	6/35.3
Antihyperlipidemic agents (count/%)	199/42.3	128/39.1	38/51.4	22/41.5	11/64.7
Antihypertensive agents (count/%)	186/39.5	112/34.3	38/51.4	26/49.1	10/58.8

Data are presented as means ± standard deviations or medians (interquartile ranges).

*SGLT2* sodium-glucose cotransporter 2, *GLP-1RAs* glucagon-like peptide-1 receptor agonists.

**Table 2:** Physical activity, body composition and physical capabilities of participants and fall incidence rate.

Parameter	All *n* = 471	Neither *n* = 327	SGLT2 inhibitors *n* = 74	GLP-RAs *n* = 53	Combination *n* = 17
Physical activity					
Steps (steps/day)	6377 ± 3409	6408 ± 3427	6677 ± 3190	6222 ± 3067	4958 ± 3276
Moderate-to-vigorous physical activity time (min/day)	11.8 (5.4–23.2)	12.1 (6.7–23.2)	14.6 (6.3–25.4)	9.0 (4.5–18.6)	5.2 (4.2–13.5)
Body composition					
Skeletal muscle percentage (%)	37.4 ± 5.4	37.7 ± 5.3	36.9 ± 5.3	35.7 ± 6.3	37.2 ± 4.7
Body fat percentage (%)	32.9 ± 9.3	32.0 ± 9.1	34.9 ± 8.8	34.8 ± 11.3	34.1 ± 8.9
Skeletal muscle mass index (kg/m^2^)	7.3 ± 1.1	7.1 ± 1.1	7.7 ± 1.1	7.4 ± 1.2	7.3 ± 1.1
Muscle strength					
Knee extension strength (Nm/kg)	1.49 ± 0.46	1.50 ± 0.45	1.54 ± 0.47	1.43 ± 0.52	1.35 ± 0.44
Knee extension endurance (J)	817 (610–1095)	791 (586–1059)	851 (659–1215)	857 (622–1046)	779 (622–1190)
Dorsiflexion strength (ankle joint) (kgf)	32.4 ± 7.5	31.7 ± 7.2	35.5 ± 8.2	32.4 ± 7.1	34.1 ± 8.3
Toe pinch force (kgf)	3.8 (3.0–4.8)	3.8 (3.0–4.7)	4.3 (3.4–5.2)	3.6 (2.7–4.3)	4.4 (3.3–4.8)
Grip strength (kgf)	29 (22–36)	28 (21–35)	31 (24–40)	29 (22–35)	28 (24–31)
Balance capability					
Index of postural stability	1.56 (1.25–1.75)	1.56 (1.25–1.78)	1.59 (1.34–1.75)	1.45 (1.14–1.69)	1.46 (0.80–1.74)
Modified index of postural stability	0.16 (0.06–0.34)	0.16 (0.06–0.32)	0.23 (0.07–0.40)	0.12 (0.04–0.33)	0.11 (0.04–0.22)
One-leg standing time (s)	41 (12–113)	41 (15–110)	57 (16–120)	22 (9–104)	28 (8–87)
Flexibility					
Finger-to-floor distance (cm)	−2 (− 13–5)	−2 (− 12–6)	−5 (− 18–5)	−4 (− 12–3)	−6 (− 13–5)
Fall incidence rate of observation (count/%)	173/17.1	116/15.0	28/22.4	20/22.5	9/36.0

Data are presented as means ± standard deviations or medians (interquartile ranges).

*SGLT2* sodium-glucose cotransporter 2, *GLP-1RAs* glucagon-like peptide-1 receptor agonists.

**Table 3:** Discrete-time logistic model 1–4 for extracting risk factors for falls.

	Model 1	Model 2	Model 3	Model 4
Sodium-glucose cotransporter 2 inhibitors/neither	1.80 (1.10–2.92)*	2.02 (1.21–3.33)**	2.02 (1.21–3.33)*	1.90 (1.13–3.15)*
Glucagon-like peptide-1 receptor agonists/neither	1.61 (0.88–2.84)	1.58 (0.86–2.81)	1.58 (0.86–2.81)	1.69 (0.89–3.09)
Combination of sodium-glucose cotransporter 2 inhibitors and glucagon-like peptide-1 receptor agonists/neither (*p* for interaction)	2.89 (1.27–6.56)* (0.992)	2.93 (1.27–6.76)* (0.879)	2.93 (1.27–6.76)* (0.877)	3.13 (1.29–7.55)* (0.966)
Fall history at admission	2.26 (1.57–3.26)**	2.13 (1.47–3.09)**	2.13 (1.47–3. 09)**	2.19 (1.50–3.20)**
Gender (female/male)	1.73 (1.03–2.89)*	1.39 (0.79–2.44)	1.39 (0.79–2.44)	1.53 (0.86–2.71)
Age	1.02 (1.01–1.04)**	1.02 (1.00–1.04)*	1.02 (1.00–1.04)*	1.02 (1.00–1.04)
Height	1.00 (0.97–1.03)	1.01 (0.98–1.04)	1.01 (0.98–1.04)	1.01 (0.98–1.04)
Body mass index	1.02 (0.99–1.06)	1.02 (0.98–1.07)	1.02 (0.97–1.07)	1.03 (0.98–1.08)
Grip strength		0.99 (0.95–1.02)	0.99 (0.95–1.02)	0.98 (0.95–1.02)
Fasting serum C peptide		1.00 (0.85–1.18)	1.00 (0.85–1.18)	0.98 (0.83–1.15)
Knee extension strength		0.80 (0.45–1.40)	0.80 (0.45–1.40)	0.85 (0.48–1.50)
Dorsiflexion strength		0.98 (0.95–1.02)	0.98 (0.95–1.02)	0.98 (0.94–1.01)
Retinopathy		1.50 (0.77–2.81)	1.50 (0.77–2.81)	1.53 (0.78–2.88)
Insulin		0.85 (0.58–1.25)	0.85 (0.58–1.25)	0.81 (0.55–1.19)
Sulfonylureas and glinides		0.81 (0.45–1.41)	0.81 (0.45–1.41)	0.72 (0.39–1.27)
Biguanides		0.90 (0.63–1.31)	0.90 (0.62–1.31)	0.83 (0.57–1.22)
Weight change			1.00 (0.97–1.03)	1.00 (0.96–1.03)
Dipeptidyl peptidase-4 inhibitors				1.36 (0.96–2.03)
α-Glucosidase inhibitors				1.18 (0.63–2.12)
Thiazolidinedione				1.58 (0.77–3.10)

Value: odds ratio (95% confidence interval).

*p < 0.05, **p < 0.01.

**Fig. 2:**
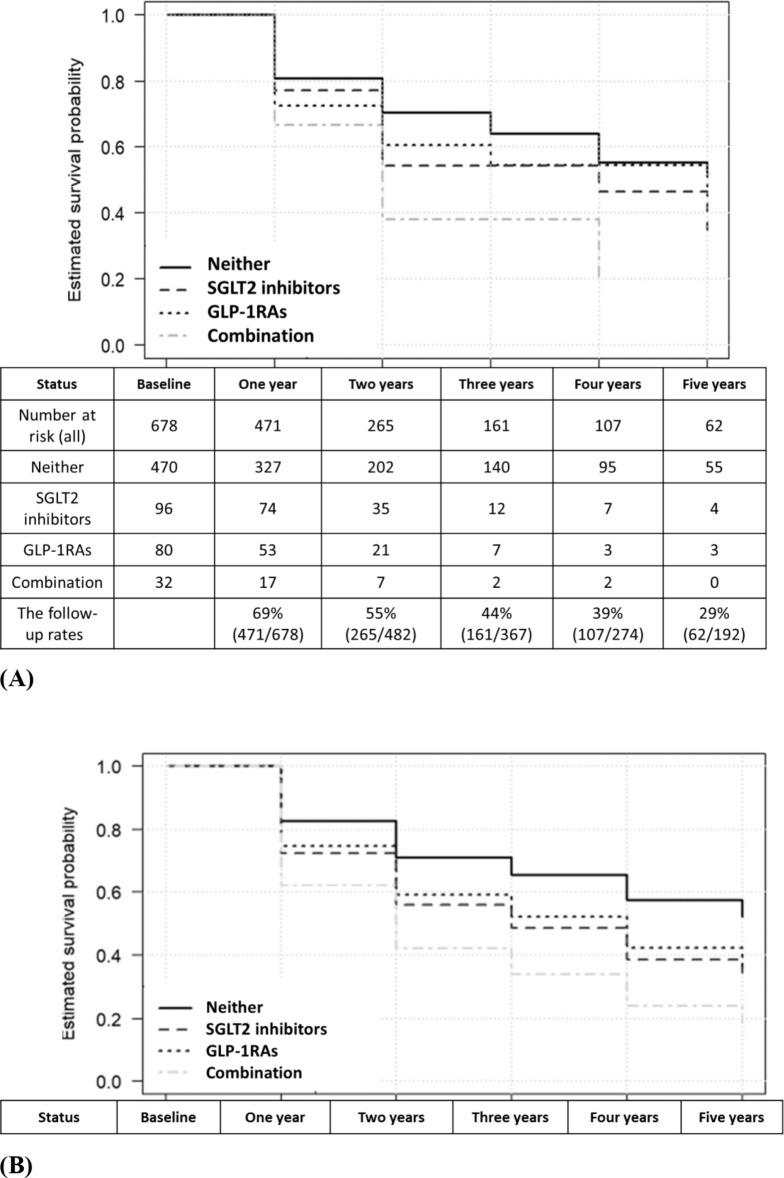
Five-year (**A**) nonadjusted and (**B**) Model 1–adjusted survival curves for four groups, namely sodium-glucose cotransporter 2 (SGLT2) inhibitors only, glucagon-like peptide-1 receptor agonists (GLP-1RAs) only, SGLT2 inhibitors + GLP-1RAs, and neither of the two types of drug.

Figure 2A,B show the unadjusted and adjusted (based on Model 1) survival curves, respectively, for four groups, namely SGLT2 inhibitors only, GLP-1RAs only, SGLT2 inhibitors + GLP-1RAs, and neither of the two types of drugs. The odds ratio for falls in the case of combined drug use, i.e., 2.89 (1.27–6.56) significantly exceeded that observed for the use of neither drug.

## Discussion

A prospective survey on falls in persons with type 2 diabetes was conducted at 1-year intervals for up to 5 years after discharge to investigate the association of SGLT2 inhibitor and GLP-1RA intake on fall incidence. The intake of SGLT2 inhibitors was identified as an independent risk factor for falls, and the combined intake of SGLT2 inhibitors and GLP-1RAs resulted in a higher fall incidence rate than the use of either drug type on its own. The association between SGLT2 inhibitor use and falls remained an independent risk factor for falls even after adjusting for known risk factors for falls, such as history of falls, skeletal muscle mass, muscle strength, and retinopathy. This finding may provide important implications for the treatment strategy of diabetes in the elderly.

SGLT2 inhibitors induce weight loss through energy loss due to urinary glucose excretion, leading to a reduction in body fat, lean body, and skeletal muscle masses^19^. Decreases in skeletal muscle mass and strength are associated with falls^12,20^. In addition, SGLT2 inhibitors increase urine volume through osmotic diuresis, thus decreasing fluid volume and increasing urinary frequency^21,22^. Frequent urination, especially nocturia, increases the risk of falls^22^. A retrospective study showed that 19% of participants who started taking SGLT2 inhibitors discontinued their intake because of frequent urination^23^. Fluid volume depletion is a risk factor for (orthostatic) hypotension^24^, thus increasing the risk of falls^25,26^. The odds ratio for orthostatic hypotension due to SGLT2 inhibitor intake was reported as 1.24 (95% confidence interval: 1.08–1.43) compared with the control group^27^. Thus, the increased risk of falls due to the intake of SGLT2 inhibitors was ascribed to the concomitant decrease in skeletal muscle mass, increase in urinary frequency, and decrease in blood volume.

GLP-1RA intake was not identified as a significant fall predictor, possibly because most participants used GLP-1RAs with a low weight-reducing effect, such as dulaglutide, liraglutide, exenatide XR, and lixisenatide, which did not strongly influence the risk of falls. Only seven participants used semaglutide, which has a strong weight-reducing effect. However, no interaction between SGLT2 inhibitor and GLP-1RA intakes was observed. The odds ratio of falls observed for the combined use of these drugs exceeded those observed for each drug individually. Given that the same trend was previously reported for weight loss^28^, the increased risk of falls observed for the combined use of the above drugs may be due to the concomitant increase in muscle weakness. Semaglutide and tirzepatide have strong weight loss effects and may further increase the risk of falls, which is a topic of future investigations. Herein, no significant relationship was observed between the occurrence of falls and weight changes. As the current study did not specify the timing of weight measurements, e.g., time of day or before/after meals, and relied on self-reported values, the weight changes could be inaccurate.

SGLT2 inhibitors and GLP-1RAs are recommended and often administered together in persons with type 2 diabetes to prevent cardiovascular and renal events, and promote weight loss^29^. The increased risk of falls associated with the intake of SGLT2 inhibitors alone, and in combination with GLP-1RAs, suggests that caution should be exercised when these drugs are administered to sarcopenia-affected persons with type 2 diabetes. However, the administration of dapagliflozin to patients with heart failure or chronic kidney disease was reported to be beneficial regardless of frailty^30,31^. In this context, the increased risk of falls should not prevent the use of SGLT2 inhibitors in persons with sarcopenia or frailty. However, when administering SGLT2 inhibitors or GLP-1RAs to these patients, one should implement nutritional and exercise therapies to minimize muscle mass loss.

Sulfonylurea and insulin administrations were not significantly associated with falls, possibly because intensive inpatient education and optimization of diabetes management, including the introduction of continuous glucose monitoring for patients receiving insulin therapy, effectively reduced the risk of hypoglycemia.

In summary, the effects of SGLT2 inhibitors and GLP-1RAs on falls in persons with type 2 diabetes were investigated by prospectively following up hospitalized elderly and nonelderly individuals with type 2 diabetes for up to 5 years after discharge. The use of SGLT2 inhibitors significantly increased the risk of falls, whereas that of GLP-1RAs had an insignificant effect. Furthermore, no interaction between the two drugs was observed, and the risk of falls further increased upon their combined administration. Our findings suggest the need to (i) consider the increased risk of falls in the case of combined therapy and (ii) provide fall-prone persons receiving such therapy with an appropriate diet and exercise.

The limitations of our study include the follow-up rate: for cohort studies, an 80% follow-up rate is desirable, whereas our first follow-up rate was 69%. Therefore, a selection bias could not be ruled out. Moreover, as questionnaires were mailed every year after discharge, a recall bias is possible. Previous studies found that 13% of participants who confirmed experiencing falls could not recall having fallen at the end of the study (i.e., after 12 months)^32^. Therefore, the fall rate could have been underestimated. Furthermore, the participants were hospitalized with poorly controlled blood glucose levels, i.e., our results may not necessarily apply to persons with type 2 diabetes with stable glycemic control in an outpatient setting. Additionally, although this research commenced in February 2014, SGLT2 inhibitors were not introduced in Japan until April 2014. Over the course of the study period, the prescription trends for antidiabetic medications shifted due to accumulating evidence supporting the efficacy of SGLT2 inhibitors and GLP-1RAs. Consequently, participants’ treatment regimens may have changed during follow-up. However, as our study did not track medication changes throughout the observation period, potential treatment-related biases cannot be excluded. Finally, this study presents results from a single center, and the validity of our findings should therefore be confirmed for larger and more diverse populations, including those with different racial backgrounds.

## Methods

### Study design and participants

A questionnaire surveying falls over a duration of 5 years was carried out on 678 persons with type 2 diabetes who were totally independent in walking and daily living activities but had been admitted to the University of Tsukuba Hospital because of poor glycemic control between February 2014 and December 2021. The exclusion criteria were (i) vitreous hemorrhage or retinal detachment, (ii) New York Heart Association functional class of II or higher, (iii) malignancy under treatment, (iv) intake of glucocorticoids, Cushing’s syndrome, or acromegaly, (v) postgastrectomy, (vi) inability to walk independently without assistive devices, (vii) nondiabetic neuropathy, and (viii) difficulty in understanding instructions.

This study was approved by the Ethics Committee of the University of Tsukuba Hospital (approval number: H27-31) and conducted according to the Declaration of Helsinki. Written informed consent was obtained from all participants.

### Follow-up of falls and body weight

Fall was defined as “coming in contact with the ground (or floor) from a standing or sitting position with a body part other than the foot in contact with the ground (floor surface) against the patient’s intention”^33^. The reliability of surveying the occurrence of falls over a previous 1-year period using the recall method was confirmed previously^32^. A fall history for the previous year was obtained on hospital admission. Each year after their discharge, the study participants were mailed a questionnaire asking about the number of falls (never, once, twice, or more) experienced within the previous 1-year period and their current weight.

### Clinical data acquisition, laboratory tests, and physical function assessment

Clinical data acquisition, laboratory tests, and physical function assessments were performed as described elsewhere^12^. Sociodemographic information, history of falls for the previous year at admission, medical history, anthropometric data, and information on medications administered at discharge were collected. Body composition was assessed by bioelectrical impedance analysis (InBody 720, InBody Japan, Tokyo, Japan). Body mass index (BMI) was calculated by dividing weight (kg) by the square of height (m^2^). Skeletal muscle mass index was calculated by dividing limb skeletal muscle mass (kg) by the square of height (m^2^).

Diabetic retinopathy was assessed by ophthalmologists. Diabetic polyneuropathy was diagnosed based on two or more of the following four criteria from The Diabetic Neuropathy Study Group in Japan and Michigan Neuropathy Screening Instrument: (i) decreased vibration perception using a 128-Hz tuning fork at the bilateral medial malleoli (< 10 s), (ii) loss of tactile sensation using a 10-g monofilament at the bilateral foot, (iii) decreased or absent bilateral Achilles tendon reflex, and (iv) numbness, pain, paresthesia, or hypoesthesia in the bilateral lower limbs or feet. Peripheral artery disease was diagnosed when the ankle-brachial index for either of the lower limbs was less than 0.9. Cardiac autonomic neuropathy was diagnosed when the variation coefficient of RR intervals at rest was below 2%. Diabetic nephropathy corresponded to urinary albumin excretion rates of ≥ 30 mg/day.

Blood samples were collected in the morning after an overnight fast within 3 days after admission to measure the plasma levels of glucose and serum levels of total cholesterol, high-density lipoprotein cholesterol, triglycerides, low-density lipoprotein-cholesterol, creatinine, C-peptide, and HbA1c. The glomerular filtration rate was estimated using an equation modified for the Japanese population^34^.

Physical activity during hospitalization was assessed using an accelerometer (Mediwalk, Terumo, Tokyo, Japan). Knee extension strength and knee extension endurance on the dominant foot side were measured using an isokinetic dynamometer (Biodex System 3, Sakai Medical, Tokyo, Japan). The dorsiflexion strength of the ankle joint was determined using a hand-held dynamometer (µTAS F-1, Anima, Tokyo, Japan). Toe pinch force was assessed using a pinch force dynamometer (Checker-kun, Nissin Sangyo, Saitama, Japan). The grip strength of the dominant hand was assessed using a Smedley analog grip strength tester (ST100T-1780, Toei Light, Tokyo, Japan). Truncal flexibility was assessed by measuring the finger–floor distance.

Balance capability was assessed using the one-leg standing time with eyes open and index of postural stability^35^. The latter index was determined using a gravicorder (GP-6000, Anima, Tokyo, Japan), and the modified index of postural stability was measured while the participant stood with eyes closed on a gravicorder covered with foam rubber^35^.

### Statistical analysis

The sample size was determined by considering the number of cases in prior studies on falls and number of participants that could be annually enrolled in the present study. Participants with missing data (31 persons) were excluded from the analysis.

Continuous variables were checked for normal distribution using the Shapiro–Wilk test and expressed as means ± standard deviations or medians (25th and 75th percentiles) based on the distribution.

Four discrete–time logistic models^36,37^ were constructed to extract fall risk factors. Model 1 considered gender, age, height, BMI, SGLT2 inhibitors, GLP-1RAs, interaction of SGLT2 inhibitors and GLP-1RAs, and fall history at admission. Model 2 considered the factors of Model 1 plus insulin, sulfonylureas/glinides, metformin, grip strength, fasting serum C-peptide level, knee extension strength, dorsiflexion strength, and proliferative diabetic retinopathy. Model 3 considered the factors of Model 2 plus weight change. Model 4 considered the factors of Model 3 plus dipeptidyl peptidase-4 inhibitors, α-glucosidase inhibitors, and thiazolidinediones.

All statistical analyses were conducted using R software (version 4.3.2; R Core Team, Vienna) and SPSS software (version 24.0; IBM Japan). Significance levels for all statistical tests and confidence levels for all confidence intervals were set at 0.05 and 95%, respectively.

## Data Availability

The datasets used and/or analysed during the current study available from the corresponding author on reasonable request.
